# Effect of the Compartmentalization Into Liposome's Cavity on the Relaxation Times of F^−^ and PF_6_^−^ Anions

**DOI:** 10.1002/nbm.70084

**Published:** 2025-06-19

**Authors:** Diana Costanzo, Francesca Garello, Silvio Aime, Enzo Terreno

**Affiliations:** ^1^ Molecular & Preclinical Imaging Center, Department of Molecular Biotechnology and Health Sciences University of Turin Turin Italy; ^2^ IRCCS SDN SynLab Naples Italy

**Keywords:** ^19^F MRI, drug delivery, drug release, liposomes, ultrasound

## Abstract

The entrapment of fluoride (F^−^) and hexafluorophosphate (PF_6_^−^) anions within liposome inner cavities significantly alters their magnetic resonance properties, offering potential advancements in imaging technologies. This study addresses the need for improved MRI contrast agents, particularly those enabling precise monitoring of drug delivery systems. By leveraging the unique interaction between these anions and the liposomal membrane, we investigate their effects on nuclear magnetic relaxation.

Specifically, we observe that both longitudinal (T_1_) and transverse (T_2_) relaxation times of the nuclei associated with the encapsulated anions are substantially shortened. This relaxation enhancement is dependent on vesicle size, being more pronounced for smaller liposomes, and varies with anion type and concentration. Notably, PF_6_^−^ induces a greater reduction in T_1_ and T_2_ relaxation times compared to F^−^. The observed effects are attributed to the dynamic interactions between the anions and the liposomal bilayer, which are modulated by the vesicle's physicochemical properties.

The results reveal a striking two‐order magnitude decrease in the ^19^F T_1_ of liposomes loaded with PF_6_^−^, demonstrating their potential utility as sensitive MRI reporters.

This work underscores the broader implications of tailoring liposome compositions for specific biomedical applications. The study not only advances the understanding of liposome‐anion interactions but also establishes a pathway for the development of novel contrast agents with high sensitivity and specificity, bridging the gap between material science and clinical imaging innovations.

## Graphical abstract

The crowding of PF_6_^−^ and F^−^ anions inside the aqueous cavity of phosphatidylcholine containing liposomes yields a dramatic ^19^F relaxation enhancement. This property makes these nanoparticles excellent ^19^F MRI systems for reporting on delivery/release of liposome payloads. The interactions with the liposomal membranes cause an overall slowdown of the anions' motions.

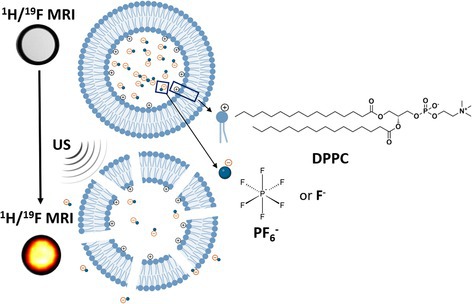

**Abbreviations**
CEST: chemical exchange saturation transfer
DPPC: 1,2‐Dipalmitoyl‐sn‐glycero‐3‐phosphocoline
DSPE‐PEG2000: 1,2‐Distearoyl‐sn‐glycero‐3‐phosphoethanolamine‐N‐(methoxy (polyethyleneglycol)‐2000 ammonium salt
NAV: number of averages
NPs: nanoparticles
PDI: polydispersion index
PFCs: perfluorocarbons
PFPEs: perfluoropolyethers
RES: reticuloendothelial system
RF: rare factor
TE: echo time
TR: repetition time
US: ultrasound

## 1 Introduction

The use of heteronuclei (i.e., different from ^1^H) in magnetic resonance imaging (MRI) arises from the need to significantly increase the contrast‐to‐background ratio that can be quite low in ^1^H‐MRI detection due to the high content of water in the body [1, 2, 3, 4, 5, 6, 7]. First reported in the early 1970s, ^19^F MRI is becoming a consolidated modality that relies on the advantageous NMR properties of the fluorine‐19 isotope. Compared to ^1^H, the ^19^F nucleus has slightly lower sensitivity (83%) and Larmor frequency value (94%), thus opening the possibility to use the same scanners as for standard ^1^H MRI, with just small adjustments [8, 9, 10, 11].

^19^F has a gyromagnetic ratio very close to that of proton (i.e., Larmor frequency of 40.08 vs. 42.58 MHz/T), and shares with ^1^H a natural abundance of 100% and a nuclear spin of ½. However, there are no MRI‐detectable endogenous ^19^F spins in soft tissues, since fluorine is mainly found in teeth and bones, where the nuclei have very short T_2_ relaxation times that definitively hamper the detection of their MR signals. Hence, the absence of background signals makes the use of exogenous ^19^F probes well‐suited for quantitative MR investigations [12, 13, 14, 15].

Despite these advantages, one should note that to obtain enough ^19^F signal intensity during a reasonable experimental time, quite high concentrations of fluorinated probes are required [16]. One way to overcome this sensitivity issue without affecting too much the image spatial resolution has dealt with the increase in the number of chemically equivalent fluorine atoms on the probe [11].

Another drawback is associated with the quite long ^19^F relaxation times, typically ranging between 0.5 and 3 s for small diamagnetic compounds, which imply long image acquisition times to obtain a sufficient signal‐to‐noise ratio (SNR) in the acquired MR images. To bypass this point, fluorinated probes can be conjugated to paramagnetic complexes. To this end, various factors must be considered, among which are the distance between fluorine and the paramagnetic center, the symmetry of the molecule, and the dynamic properties of the probe [16, 17, 18, 19, 20].

At present, among the different types of fluorinated probes that have been developed [21], perfluorocarbons (PFCs) and fluorinated polymers, especially perfluoropolyethers (PFPEs), are the most employed at the preclinical/clinical levels for ^19^F MRI applications, given the high mass density of ^19^F atoms [10, 12]. However, there is a limitation due to the high hydrophobicity of these fluorinated compounds, which hampers the water solubility of the resulting probes. Nevertheless, this limitation has been addressed by formulating ^19^F‐rich nanoemulsions that are colloidal suspensions of PFC/PFPE droplets, stabilized with a surfactant [13]. These ^19^F‐rich nanoemulsions have been largely tested in cell labeling experiments, both for in vivo and ex vivo cell labeling. In vivo cell labeling is achieved by direct intravenous injection of the nanoemulsions, which are then taken up by immune system cells both in reticuloendothelial system (RES) organs and at the disease inflammation sites. For ex vivo cell labeling, instead, the cells are co‐incubated with the specifically designed nanoemulsion and then injected or transplanted into the subject [22]. Recently, nanoemulsions formulated with different fluorinated compounds, characterized by distinct ^19^F chemical shifts, have been successfully employed for multispectral ^19^F MRI to identify spatially and temporally distinct cell populations [23]. The extremely large spectral range of fluorinated molecules (> 350 ppm) and the possibility of quantifying them separately make multispectral ^19^F MRI extremely appealing. The probes tested for multispectral ^19^F MRI can be based on PFCs, small fluorine‐containing molecules, fluorinated ionic liquids, and inorganic fluoride nanoparticles.

Thinking about alternative nanocarriers for fluorinated molecules, liposomes, dendrimers, inorganic, and polymeric nanoparticles (NPs) can be considered [24]. In this regard, liposomes represent an excellent choice due to their remarkable chemical versatility and good clinical translatability. In principle, liposomes can encapsulate both hydrophilic and hydrophobic agents, making them excellent systems to address the above‐mentioned sensitivity issue and to fully exploit the benefits of ^19^F MRI. Dewitte et al., for example, developed PFCE‐loaded liposomes for dendritic cell targeting via ^19^F MRI [25].

Moreover, previous studies aimed at developing temperature‐sensitive procedures demonstrated the potential of using liposomes entrapping both PF_6_^−^ (for ^19^F detection) and a paramagnetic chemical exchange saturation transfer (CEST) agent (Tm‐HPDO3A, for ^1^H detection). The activation of the combined response of the two reporters in response to local temperature increases allowed us to nicely mimic and quantify the drug release from the nanovesicle [26]. As these liposomes displayed a discrete MRI contrast, we aimed to prepare ultrasound‐responsive PF_6_^−^‐loaded liposomes without co‐loading other imaging probes in order to evaluate their potential in ^19^F MRI experiments on imaging drug delivery and release processes.

## 2 Experimental

### 2.1 Chemicals

1,2‐Dipalmitoyl‐sn‐glycero‐3‐phosphocoline (DPPC) and 1,2‐Distearoyl‐sn‐glycero‐3‐phosphoethanolamine‐N‐(methoxy (polyethyleneglycol)‐2000 ammonium salt (DSPE‐PEG2000) were purchased from Avanti Polar Lipids Inc. (Birmingham, AL, USA). All the other chemicals were purchased from Sigma‐Aldrich (St. Louis, MO, USA).

### 2.2 Liposome Formulation

Liposomes were formulated as follows: DPPC/Cholesterol/DSPE‐PEG2000 in a molar ratio of 74:20:6. The total amount of membrane components was calculated to have a final concentration in the hydration solution of 20 mg/mL. Phospholipids were dissolved in chloroform, and the solvent was evaporated under vacuum through a rotary evaporator (Heidolph Instruments, Germany). The thin lipid film was hydrated at 55°C by adding 1.5 mL of NaPF_6_ dissolved in bidistilled water at concentrations of 50, 100, or 150 mM. To achieve a final osmolarity of 300 mOsm, HEPES/NaCl buffer was added to the 50‐ and 100‐mM NaPF_6_ solutions to adjust their osmolarity accordingly. The resulting suspensions were extruded through polycarbonate filters of decreasing pore diameters (from 800 to 100 nm, LIPEX extruder, Northern Lipids Inc., Burnaby, BC, Canada). Next, liposomes were dialyzed overnight against isotonic HEPES/NaCl buffer using a dialysis cellulose membrane with a molecular weight cutoff of 12,400 Da to separate the nonencapsulated compound.

Blank liposomes were prepared as described above, using 1.5 mL of isotonic HEPES/NaCl buffer as the hydration solution. A batch of larger liposomes (hydrodynamic diameter ~400 nm) was prepared using the same procedure, except that extrusion was performed twice through 800‐nm pore‐size filters and twice through 400‐nm pore‐size filters. A batch of smaller liposomes (hydrodynamic diameter ~70 nm) was also prepared using the same procedure, adding two extrusion steps through 50‐nm pore‐size filters.

### 2.3 Liposome Characterization

The hydrodynamic diameter and polydispersion index (PDI) of the liposomes were measured by dynamic light scattering (DLS, Zetasizer Nano‐ZS, Malvern, UK).

Quantification of the PF_6_^−^ entrapped in the liposomes was performed by ^19^F NMR spectroscopy at 7 T (7‐T MRI scanner, Bruker Avance NEO, vertical bore orientation equipped with a 40‐mm ^1^H/^19^F volume transmit‐receive probe, operating at frequencies of 300 MHz for ^1^H and 282.38 MHz for ^19^F measurements), using trifluoroacetamide as reference.

Concentration of liposomes [*lipo*] in the different suspensions was calculated using the following formula:

$$\left[\text{lipo}\right]=\frac{{\left[F\right]}_{\mathrm{tot}}}{{\left[F\right]}_{\text{lipo}}\;x\;V_{\text{lipo}}\;x\;N_{\mathrm{Av}}}$$

where [*F*]_*lipo*_ is the nominal concentration of PF_6_^−^ or F^−^ inside the liposomes, assumed to correspond to the fluorine concentration in the hydration solution; *N*_*Av*_ is Avogadro's number; and *V*_*lipo*_ is the internal volume of a single liposome, calculated as:

$$V_{\text{lipo}}=\frac{4}{3}\;\pi \;x\;r_{\text{intra}}^{3}$$

where *r*_*intra*_ is the internal radius of the liposome, obtained by subtracting 5 nm (the average bilayer thickness) from the hydrodynamic radius measured by DLS.

Encapsulation yield was calculated according to the following formula:

$$\%\text{Yield}=\frac{{\left[F\right]}_{\mathrm{tot}}\;\times V_{\text{post}-\text{dyalisis}}}{{\left[F\right]}_{\text{hydration}}\times V_{\text{hydration}}}x100$$

where [F]_hydration_ is the concentration of fluorine in the hydration solution, and [F]_tot_ was defined above. Further information regarding the equation used to estimate liposome concentration can be found in the Supporting Information.

### 2.4 ^19^F and ^31^P NMR Spectra of NaPF_6_

^19^F NMR spectrum of free PF_6_^−^ solutions was acquired at 7 T, with a relaxation delay of 25 s and 48 scans.

^31^P spectra of free PF_6_^−^ solutions were acquired at 14 T using a high‐resolution NMR spectrometer (600‐MHz Bruker Avance III spectrometer), in a 5‐mm BBI probe, with a relaxation delay of 1 s and one scan.

### 2.5 Ultrasound‐Triggered Release of PF_6_^−^

The release of PF_6_^−^ from liposomes was triggered by applying ultrasounds (Bandelin Sonopuls HD 2070 Ultrasonic Homogeniser, sonication power 75%) to a suspension of liposomes hydrated with PF_6_^−^150 mM. The release was triggered by immersing the ultrasonic probe in the liposome suspension (volume 800 μL). Variable sonication times were used (10, 20, 30, 45, 60, and 120 s). After sonication, the suspensions (except for the sample sonicated for 120 s) were dialyzed overnight using a dialysis cellulose membrane with a molecular weight cutoff of 12,400 Da, against isotonic HEPES/NaCl buffer to separate the released fluorine‐containing species. Quantification of PF_6_^−^ or F^−^ after dialysis was performed using trifluoroacetamide as the standard reference, as previously reported.

### 2.6 T_1_ and T_2_ Relaxation Times Measurements

^19^F and ^31^P T_1_ and T_2_ relaxation times of PF_6_^−^ (free or encapsulated into liposomes) were determined using conventional inversion recovery (IR) and Carr‐Purcell‐Meiboom‐Gill (CPMG) sequences, respectively. Data were recorded using a Bruker Avance 7‐T MRI scanner and analyzed using Bruker TopSpin 4.0.8 software. The following acquisition parameters were used: for the IR sequence: relaxation delay 25 s, number of points = 16, 24 scans. For the CPMG sequence, the parameters were: relaxation delay 25 s, number of points = 18, 48 scans.

Variable temperature ^19^F T_1_ and T_2_ measurements were run between 25°C and 40°C for liposomes containing PF_6_^−^150 mM and for free PF_6_^−^ solutions (150 mM). The temperature of the phantom was measured before and after each scanning session using methanol chemical shift as temperature control.

### 2.7 Imaging Experiments

^1^H/^19^F MRI was performed on a Bruker Avance 7‐T MRI. Suspensions of liposomes, hydrated with PF_6_^−^150 mM, before and after US stimulation were placed into 2‐mL vials. The vials were then inserted into an agar phantom together with a water‐containing tube and imaged. For ^1^H MRI, a RARE sequence was used to acquire coronal images with the following parameters: echo time (TE) = 37.14 ms, repetition time (TR) = 3000 ms, number of averages (NAV) = 2, rare factor (RF) = 16, matrix size = 256 × 256, field of view (FOV) = 4.2 × 4.2 cm, slice thickness = 10 mm, and acquisition time = 1 min 36 s.

For ^19^F MRI, a fast low angle shot (FLASH) sequence was used. Images were acquired using the following parameters: TR = 500 ms, TE = 1.3/11.2 ms, NAV = 72, flip angle = 75°, matrix size = 32 × 32, FOV = 4.2 × 4.2 cm, slice thickness = 10 mm, and acquisition time = 14 min 25 s.

## 3 Results

### 3.1 NMR Spectra of NaPF_6_

**FIGURE 1:**
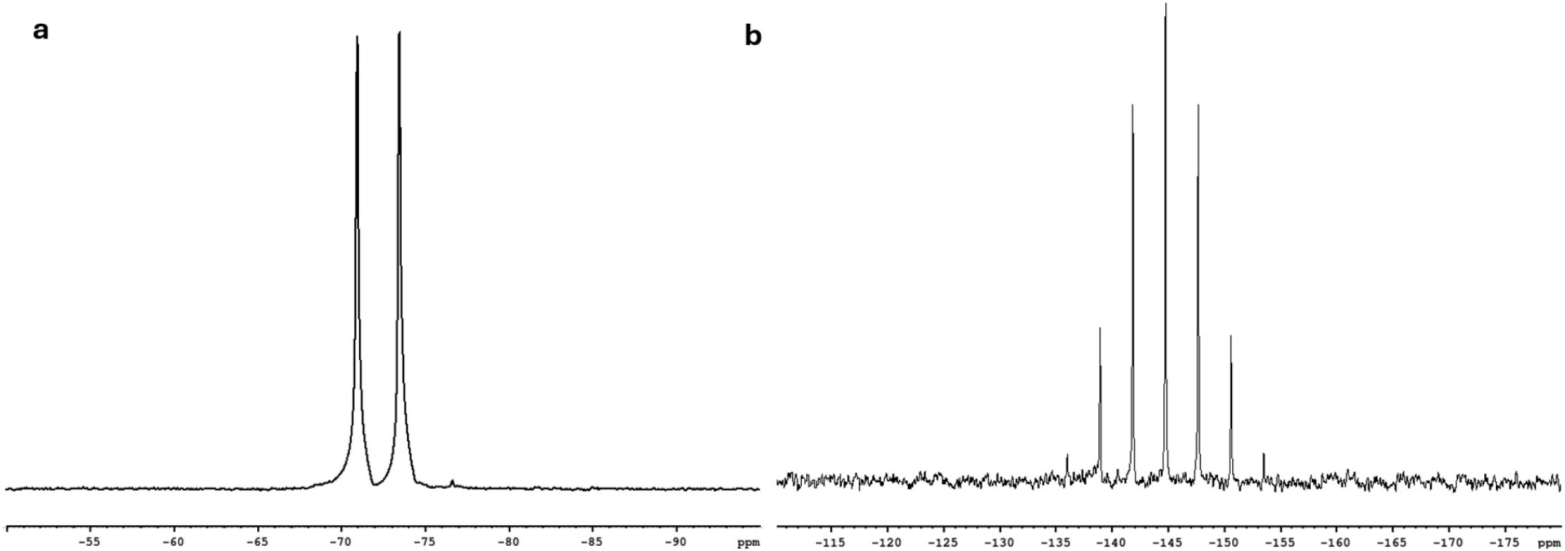
^19^F (a) and ^31^P (b) NMR spectra of NaPF_6_ in water at room temperature.

The ^19^F and ^31^P NMR spectra of PF_6_^−^ are shown in Figure 1. As expected, the ^19^F NMR spectrum showed a doublet with a ^31^P‐^19^F coupling constant of 711 Hz. The ^31^P spectrum displayed a septet with the central peak at −144.7 ppm, resulting from the coupling of the ^31^P nucleus with six fluorine atoms.

### 3.2 Liposomal Encapsulation of PF_6_^−^

By knowing the concentration of the hydration solution of PF_6_^−^ (50, 100, and 150 mM) and the size of the resulting liposomes (140 nm), it was possible to calculate the concentration of the liposomes in the obtained suspensions (see Section 2). This concentration was 97.7, 93.1, and 82.1 nM for the 150‐, 100‐, and 50‐mM hydration solutions, respectively. The PF_6_^−^ encapsulation yield ranged from 3% to 4% for all the preparations.^19^F and ^31^P NMR spectra of NaPF_6_ encapsulated in liposomes (data not shown) were fully consistent with those reported in Figure 1.

### 3.3 ^19^F and ^31^P Relaxation Times of PF_6_^−^

The effect of encapsulation of PF_6_^−^ inside the aqueous cavity of liposomes on ^19^F and ^31^P relaxation times (T_1_ and T_2_) of the anion was determined at 7 T and room temperature (Table 1, Figure S1). Both T_1_ and T_2_ values of the ^19^F resonance decreased linearly as a function of the concentration of the entrapped anion in the liposome aqueous cavity prepared with hydration solutions containing the anion in the 50–150‐mM concentration range. In contrast, the relaxation times of free, nonencapsulated PF_6_^−^ were significantly longer and did not exhibit any dependence on the concentration of the anion.

**TABLE 1:** ^19^F longitudinal (T_1_) and transverse (T_2_) relaxation times values of suspensions of liposomes encapsulating different concentrations of PF_6_^−^ and free PF_6_^−^ solutions (7 T, room temperature).

[PF_6_^−^]	LipoPF_6_^−^			Free PF_6_^−^		
	50 mM	100 mM	150 mM	50 mM	100 mM	150 mM
**T**_**1**_ **(ms)**	1183 ± 67	839 ± 48	382 ± 14	3800 ± 50	3700 ± 30	3600 ± 80
**T**_**2**_ **(ms)**	33.6 ± 3.2	24.7 ± 1.3	9.4 ± 0.7	2600 ± 30	2200 ± 40	2500 ± 20

A similar effect, although less pronounced, was observed for the relaxation times of the ^31^P nucleus (Table S1 and Figure S2).

^19^F T_1_ relaxation times measurements of free and liposome‐encapsulated PF_6_^−^ were also carried out at variable temperatures. Ongoing from 25°C to 40°C, T_1_ of the free PF_6_^−^ remained almost constant. Conversely, the T_1_ values of encapsulated PF_6_^−^ showed an increase of about 70% (Figure S3). This result suggests that increasing the temperature is particularly effective in influencing the relaxation mechanisms occurring within the confined space of the liposome's inner compartment. It can be hypothesized that the motion of the anions is modulated by their transient association with moieties on the inner surface of the liposomal membrane. Higher temperatures reduce the lifetime of these interactions, resulting in an overall increase in the molecular mobility of the anions, which leads to the observed elongation of the relaxation times. This increased mobility not only affects the exchange rate between the “bound” and “free” forms inside the liposomal cavity but may also influence the local dynamics of the “bound” state itself, thereby extending the relaxation times associated with this fraction. Although the molar fractions of the “bound” and “free” states do not change significantly over this temperature range, it is plausible that minor structural alterations and a shortening of the “bound” state's lifetime contribute to the observed behavior.

**FIGURE 2:**
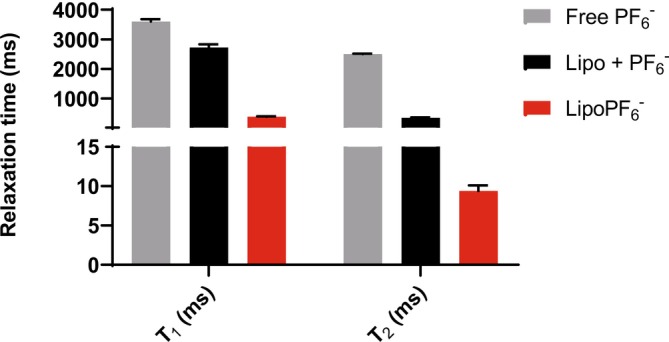
^19^F longitudinal (T_1_) and transverse (T_2_) relaxation times of liposomes encapsulating 150 mM of PF_6_^−^ (red bars) compared to the values obtained when the same concentration of the anion was added to a suspension of empty liposomes (black bars). The values were compared to those of free PF_6_^−^ solutions (grey bars).

To get further insight on whether the relaxation effects observed for the encapsulated anions have to be associated with a possible interaction of the anion with the polar heads of the components of the liposome bilayer, the ^19^F relaxation times of PF_6_^−^ were measured in a suspension of empty liposomes supplemented with 150 mM of the anion in the outer compartment (Table S2). Though both relaxation times decreased in the presence of the liposomes, the observed effect was much more attenuated with respect to what was observed in the compartmentalization of the anion in the inner aqueous core of the nanovesicle. These results are summarized in Figure 2.

### 3.4 Comparison Between PF_6_^−^ and F^−^

**FIGURE 3:**
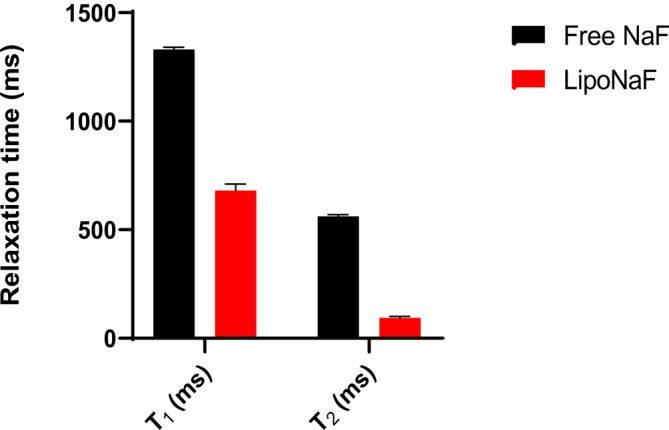
Comparison between the ^19^F relaxation times, T_1_ and T_2_ of fluoride ion free (black) or encapsulated in liposomes (red) (7 T, room temperature).

To investigate whether the compartmentalization effect observed for PF_6_^−^ is shared with other anions, liposomes hydrated with a solution of NaF (150 mM) were prepared. The encapsulation yield for this system was 2.7%. The ^19^F T_1_ and T_2_ relaxation times of these liposomes were measured at 7 T and room temperature, and the results were compared with the corresponding values for free F^−^ (at 150 mM). The results showed the shortening of both relaxation times in the liposomal sample (Table S3, Figure 3), although the compartmentalization effect for F^−^ was less pronounced than for PF_6_^−^.

**FIGURE 4:**
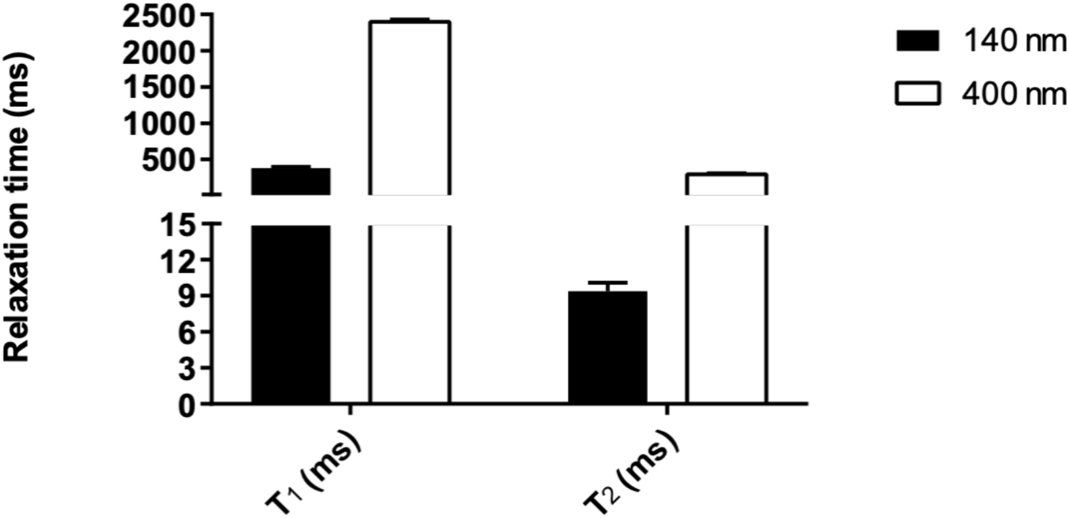
^19^F longitudinal (T_1_) and transverse (T_2_) relaxation times of PF_6_^−^‐encapsulating liposomes with a hydrodynamic diameter of 140 nm (black bars) and 400 nm (white bars).

Next, to verify whether the relaxation enhancement induced by the anion confinement in the liposome was dependent on the volume of the aqueous cavity, liposomes with larger size (400 nm vs 140 nm), hydrated with 150 mM of PF_6_^−^, were prepared. Interestingly, the ^19^F relaxation times of the anion encapsulated in the bigger liposomes showed much longer T_1_ and T_2_ values compared to the smaller liposomes, thus suggesting an important role of the vesicle size over the relaxation times of PF_6_^−^ (Table S4, Figure 4). An attempt was also made to prepare smaller liposomes (~70 nm) using the same phospholipid composition and preparation protocol. However, the resulting formulation was unstable and exhibited PF_6_^−^ leakage (Figure S4), which markedly diminished the confinement effect. Notably, in the ^19^F NMR spectrum acquired, a chemical shift difference of 0.40 ppm was observed between the centers of the ^19^F doublets corresponding to intra‐ and extra‐liposomal PF_6_^−^. This finding further supports the presence of binding interactions between the encapsulated anions and the inner surface of the vesicles.

### 3.5 Assessment of the Potential of PF_6_^−^‐Encapsulated Liposomes as ^19^F MRI Reporters of Payload Release

**FIGURE 5:**
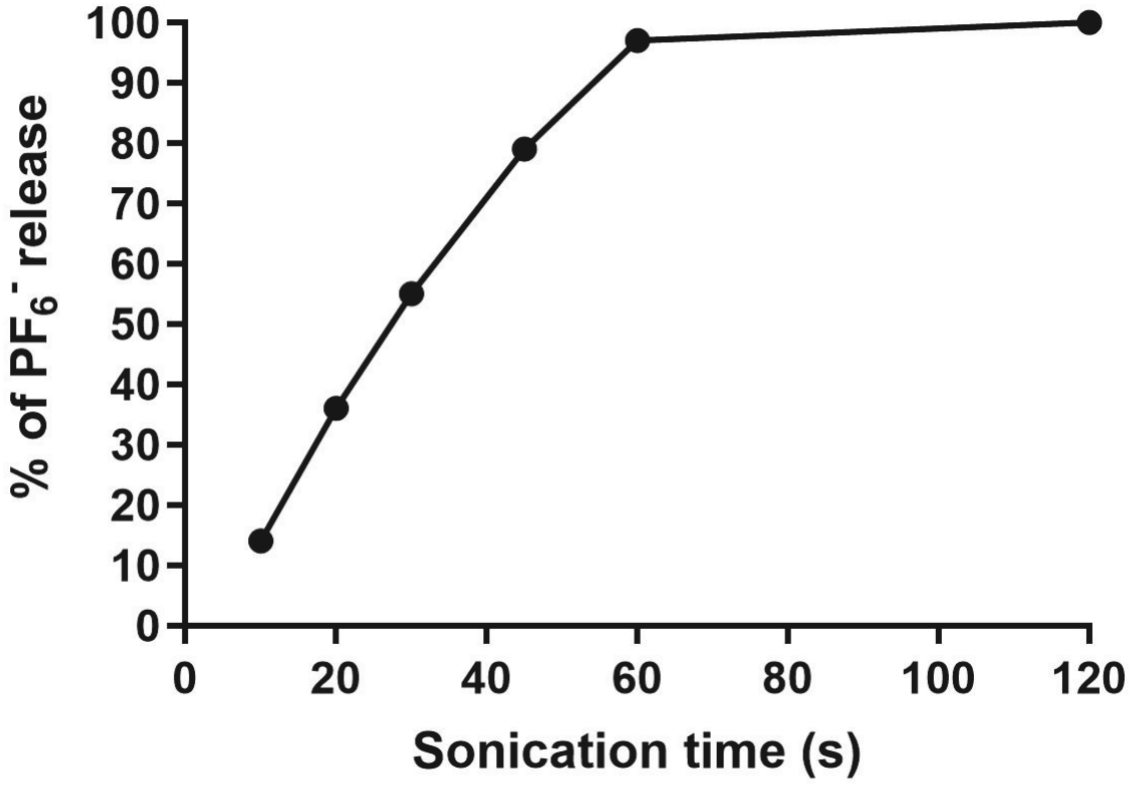
Amount (%) of PF_6_^−^ released as a function of the US irradiation time.

The remarkable effect on the ^19^F relaxation times observed when PF_6_^−^ anions are encapsulated in liposomes prompted us to test the potential of this system to develop a ^19^F MRI protocol for imaging drug delivery/release. To this end, we decided to monitor the release of PF_6_^−^ anions from liposomes subjected to ultrasound stimuli. Figure 5 shows the percentage of released PF_6_^−^ as a function of the duration of ultrasound application (see Section 2). Quantification of the released anion, after separation from the liposome‐encapsulated fraction by dialysis, was performed using ^19^F NMR spectroscopy.

**FIGURE 6:**
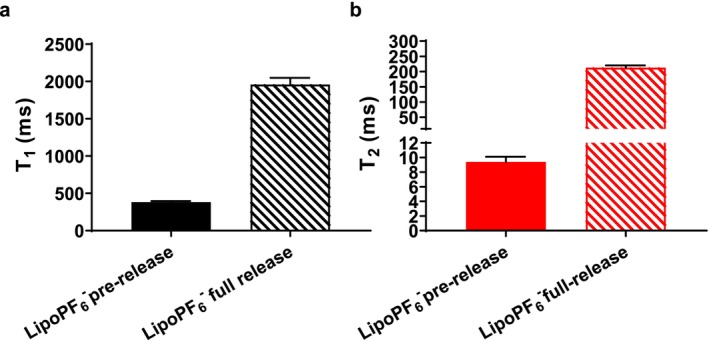
^19^F longitudinal (a) and transverse (b) relaxation times of LipoPF_6_^−^ before (full filled bars) and after (striped bars) 100% US‐induced content release.

As expected, the release of PF_6_^−^ was associated with a lengthening of both T_1_ and T_2_ relaxation times. In Figure 6, the data for the fully released sample are displayed.

To provide proof of concept for the potential application of this system as a ^19^F MRI reporter for drug delivery and release, a three‐sample phantom was prepared and imaged using ^1^H‐ and ^19^F MRI. The phantom consisted of 2‐mL tubes immersed in agar gel, filled with two suspensions of liposomes hydrated with PF_6_^−^ at 150 mM. One of these suspensions was exposed to ultrasound (US) to induce 100% release of the encapsulated anion, while the other remained unexposed. An additional tube filled with water was included in the phantom as a control.

**FIGURE 7:**
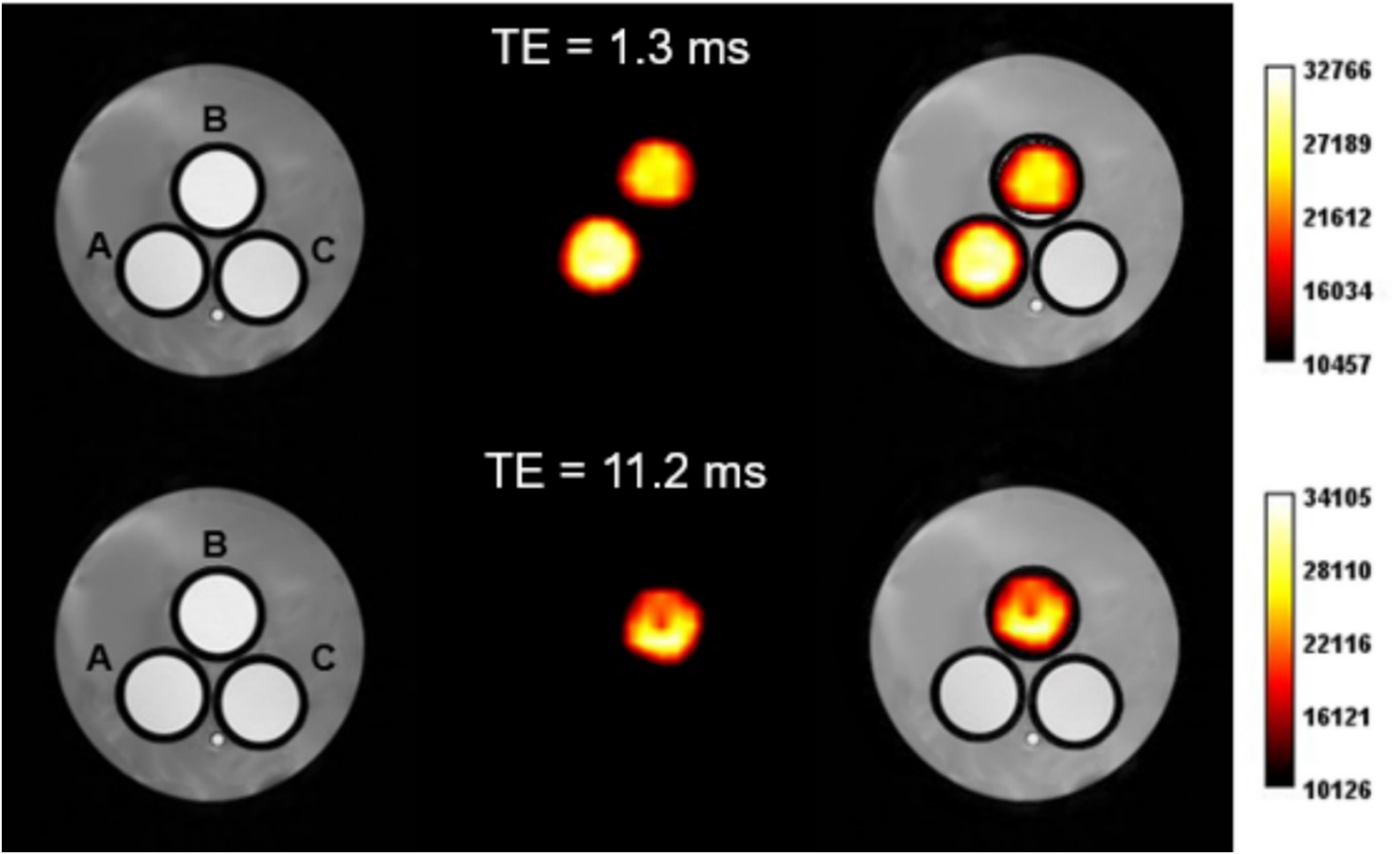
MR image of a phantom obtained using the FLASH sequence. The phantom was made of a tube containing intact liposomes encapsulating 150 mM of PF_6_^−^ (A), a tube containing the same solution after US‐induced 100% release of PF_6_^−^ (B), and a tube containing water (C). Images were acquired with the same parameters except for the echo time that was set at 1.3 ms (top) and 11.2 ms (bottom).

A FLASH sequence was employed applying two TE values, namely, 1.3 ms to visualize both ^19^F‐containing samples and 11.2 ms to visualize only the tube with the longer T_2_ value, which contained the released anion (Figure 7). In this way, the MRI signals from spins with long T_2_ values are well‐detected across a broad range of TE values, whereas spins with short T_2_ values are only detectable with very short TE values. The acquired MR images, shown in Figure 7, clearly demonstrate that by selecting an appropriate TE value, it is possible to visualize both samples (short TE) or exclusively the sample containing the released payload (long TE).

## 4 Discussion

**FIGURE 8:**
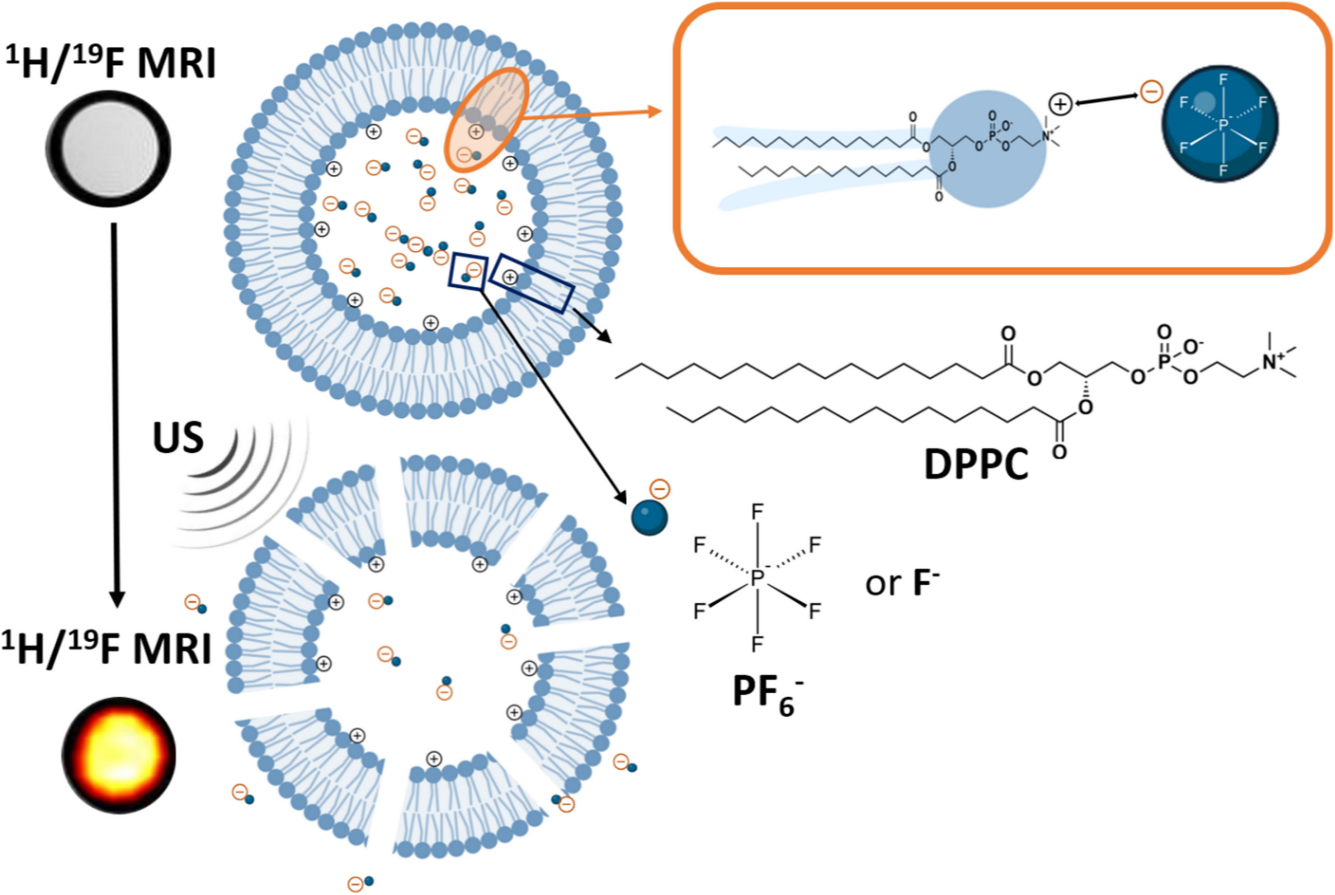
Schematic representation of the hypothesized interactions between PF_6_^−^/F^−^ and the phospholipid bilayer.

The ^19^F and ^31^P NMR relaxation times of PF_6_^−^ and the ^19^F NMR relaxation times of F^−^ anions have been under intense investigation for several decades [27]. These anions perform isotropic rotation in solution, and their fluorine and phosphorus (for PF_6_^−^ ion) spin–lattice relaxation times were found to be the sum of interactions due to spin‐rotation and dipole–dipole contributions. The comparison between the ^19^F relaxation values of PF_6_^−^ free or encapsulated in liposomes highlighted a strong decrease in T_1_ and T_2_ values when the anion is loaded inside the liposomal cavity. The experimental evidence supports the view that such an effect is the result of the dynamics of the confinement of the molecule in the limited volume of the inner liposomal cavity. One may envisage that peculiar interactions affecting the motional behavior of the anions take place in the inner liposomal cavity, resulting in a marked relaxation enhancement (Figure 8).

It was interesting to note that also when PF_6_^−^ was added to a suspension of blank liposomes, a decrease in relaxation times was observed, although definitively less significant. It appears that the cationic centers on the surface of the phospholipids may be involved in weak, reversible interactions with the anions. The large concentration of these positively charged moieties on the inner layer of the membrane allows the set‐up of multiple interactions that result in an overall decrease of the reorientational time of the anions in this microenvironment. This view is fully supported by the results obtained in liposomes encapsulating different PF_6_^−^ concentrations, indicating that a higher concentration of the ion inside the vesicles led to a stronger decrease in the relaxation times. In particular, the decrease in ^19^F‐T_1_ was 68.9%, 77.3%, and 89.4% for liposomes encapsulating 50‐, 100‐, and 150‐mM systems, respectively. The corresponding decrease in ^19^F‐T_2_ was 98.7%, 98.9%, and 99.6% at the same PF_6_^−^ concentrations. Clearly, the mechanisms operating in spin–spin relaxation are even more sensitive to the change in concentration of the anions in the inner liposomal cavity.

Additional evidence to support the view that the relaxation enhancement is strictly associated with the confinement characteristics of the aqueous cavity was found by measuring the relaxation times of liposomes of different size, loaded with the same concentration of PF_6_^−^. The results indicated a more pronounced relaxation effect in the nanovesicles of 140 nm, with the smaller inner cavity volume. Upon entrapment in the liposomal cavity, the fluoride anion exhibits a similar behavior, albeit with lower efficiency, showing a 60% and 86% reduction in T_1_ and T_2_ relaxation times, respectively. The presence of six fluorine atoms in PF_6_^−^ increases the likelihood of binding interactions with positively charged centers, leading to a lengthening of its motion compared to the monoatomic fluoride anion. Another possible explanation for this difference is the delocalization of the negative charge across the six fluorine atoms in PF_6_^−^, which may influence the molecule's interaction with the liposome environment [27].

The pronounced decrease in ^19^F relaxation times observed in this study is particularly significant for ^19^F MRI applications. Previously, a reduction in T_1_ by an order of magnitude was only attainable through the co‐encapsulation of paramagnetic species [28, 29]. In parallel to T_1_ reduction, the confinement also leads to a shortening of T_2_, which can adversely affect imaging by accelerating signal decay. However, in this work, the T_2_ shortening was intentionally leveraged to differentiate between intact and released liposomes. Additionally, this potential limitation can be addressed using ultrashort echo time (UTE) sequences, commonly employed in iron oxide and deuterium imaging, which enable TEs as short as 8–100 μs, approximately 100‐fold shorter than those used in standard clinical MRI. Variable temperature experiments on free PF_6_^−^ solutions were in line with those previously reported by Froix et al. [27]. However, the relaxation times of ions encapsulated within the liposomes were shorter, and their temperature dependence was strongly affected by the multiple interactions the anions have with liposomal membrane components. Notably, the relaxation effect observed for fluorine spins in PF_6_^−^ was greater than that for the ^31^P nucleus within the same molecule. Specifically, the decrease in T_1_ and T_2_ relaxation times for ^31^P was 48% and 84%, respectively.

Although it was not the first time that hydrophilic fluorinated molecules were stably encapsulated in liposomes, the previous studies mainly focused on the ability of the liposomal formulation to entrap fluorinated compounds with different chemical shifts for spectral ^19^F MRI [21]. In this study, we were able to formulate liposomes containing a hydrophilic ^19^F‐containing compound whose relaxation properties open the possibility to design a probe for imaging drug delivery and release.

The application of ultrasound to trigger the release of PF_6_^−^ from liposomes can be assessed by the measurement of ^19^F relaxation times. Upon release, both T_1_ and T_2_ relaxation times increase, reflecting the shift in the molecular environment from the confined liposomal cavity to free diffusion. Developing methods to track and monitor the fate of a drug after administration continues to be of pivotal interest. Compared to other techniques for assessing drug release from liposomes, such as optical methods or MRI with paramagnetic metal complexes, this system offers several advantages. For instance, fluorescent dyes used in optical methods have limited tissue penetration in vivo, and they are prone to photobleaching over time. Recent studies have highlighted the integration of hybrid MRI antenna systems with ^19^F MRI and hyperthermia, enabling precise control and monitoring of temperature‐sensitive drug release. This approach enhances precision and safety, particularly in targeted cancer therapies [30]. Ultrasound has already been shown to be a valuable non‐invasive tool in this context, facilitating the targeted and controlled release of drugs. This reduces potential side effects and enables real‐time monitoring of drug behavior [31, 32].

Here, the possibility to selectively visualize long and short T_2_ components just by varying the TE of the ^19^F MRI sequence used opens the possibility to use the system as a reporter of drug delivery and release.

## 5 Conclusions

The herein reported results indicate that one may pursue the relaxation enhancement of a given magnetically active species through its encapsulation in the inner aqueous cavity of liposomes. This achievement is relevant as it may pave the way for the design of improved relaxation liposome‐based agents. This study provides a proof‐of‐concept of the potential of PF_6_^−^ loaded liposomes to act as a probe for imaging both drug delivery and release using ^19^F MRI. The encapsulation of the ion within liposomes significantly affects the relaxation properties of the fluorine nuclei. The dependence of the relaxation times on the concentration of PF_6_^−^ and the size of the liposomal cavity highlights the critical role of molecular confinement within the liposome in modulating the MRI signal. Furthermore, the ability to trigger release via ultrasound stimulation and selectively visualize both the released and intact liposomes using MRI parameters underscores the potential of this system as a theranostic tool, integrating therapy and diagnostic imaging into a single platform. Future work could explore the co‐encapsulation of therapeutic agents or the incorporation in the liposomal formulation of cationic lipids, which may enhance or alter PF_6_^−^ interactions through electrostatic attraction, further tuning the system's responsiveness and efficacy.

Overall, this work introduces a promising direction for the development of liposome‐based systems that combine controlled release with noninvasive imaging capabilities.

## Supporting information


**Figure S1.** Comparison between the ^19^F relaxation times, T_1_ (a) and T_2_ (b) of PF_6_^−^ at different concentrations: nonencapsulated (grey) and encapsulated (black) in liposomes (7 T, room temperature).
**Table S1.** ^31^P T_1_ and T_2_ relaxation times of PF_6_^−^‐loaded liposomes at different concentrations and of free PF_6_^−^ 150 mM.
**Figure S2.** ^31^P T_1_ and T_2_ relaxation times of liposomes encapsulating PF_6_^−^ at different concentrations compared to a free PF_6_^−^ solution 150 mM.
**Figure S3.** ^19^F T_1_ and T_2_ measurements of free PF_6_^−^ and lipoPF_6_^−^ 150 mM at different temperatures.
**Figure S4.** ^19^F NMR spectrum of small‐sized liposomes (70 nm) acquired at 400 MHz, 298 K, 4 h after dialysis, demonstrating the simultaneous presence of both internalized (larger peaks) and released NaPF_6_.
**Table S2.** ^19^F T_1_ and T_2_ relaxation times values of free PF_6_^−^ (150 mM), liposomes encapsulating PF_6_^−^ (PF_6_^−^ inside), and liposomes added with PF_6_^−^ (PF_6_^−^ outside) (7 T, room temperature).
**Table S3.** ^19^F longitudinal (T_1_) and transverse (T_2_) relaxation times of a 150‐mM solution of fluoride ion and of a suspension of liposomes encapsulating 150 mM of fluoride ion (7 T, room temperature).
**Table S4.** ^19^F T_1_ and T_2_ relaxation times values of liposomes encapsulating PF_6_^−^ with a hydrodynamic diameter of 140 and 400 nm.

## Data Availability

The data that support the findings of this study are available from the corresponding author upon reasonable request.
