# Supplementary material for: Effect of the Compartmentalization Into Liposome's Cavity on the Relaxation Times of F− and PF6 − Anions
Source: NMR Biomed. 2025 Jun 19;38(8):e70084. doi: 10.1002/nbm.70084 (PMC12179491; doi:10.1002/nbm.70084)
Supplement: Supplementary file 1 — Figure S1. Comparison between the 19F relaxation times, T1 (a) and T2 (b) of PF6 − at different concentrations: nonencapsulated (grey) and encapsulated (black) in liposomes (7 T, room temperature). Table S1. 31P T1 and T2 relaxation times of PF6 −‐loaded liposomes at different concentrations and of free PF6 − 150 mM. Figure S2. 31P T1 and T2 relaxation times of liposomes encapsulating PF6 − at different concentrations compared to a free PF6 − solution 150 mM. Figure S3. 19F T1 and T2 measurements of free PF6 − and lipoPF6 − 150 mM at different temperatures. Figure S4. 19F NMR spectrum of small‐sized liposomes (70 nm) acquired at 400 MHz, 298 K, 4 h after dialysis, demonstrating the simultaneous presence of both internalized (larger peaks) and released NaPF6. Table S2. 19F T1 and T2 relaxation times values of free PF6 − (150 mM), liposomes encapsulating PF6 − (PF6 − inside), and liposomes added with PF6 − (PF6 − outside) (7 T, room temperature). Table S3. 19F longitudinal (T1) and transverse (T2) relaxation times of a 150‐mM solution of fluoride ion and of a suspension of liposomes encapsulating 150 mM of fluoride ion (7 T, room temperature). Table S4. 19F T1 and T2 relaxation times values of liposomes encapsulating PF6 − with a hydrodynamic diameter of 140 and 400 nm. [file NBM-38-e70084-s001.docx]

**Supplementary Information**

**Effect of the compartmentalization into liposome’s cavity on the relaxation times of F^-^ and PF_6_^-^ anions**

Diana Costanzo,^1^ Francesca Garello,^1^ Silvio Aime,^2^ Enzo Terreno^1*^

Affiliation

^1^ Molecular & Preclinical Imaging Centers, Department of Molecular Biotechnology and Health Sciences, University of Turin, Turin, Italy.

^2^ IRCCS SDN SynLab, Napoli, Italy.

*Corresponding Author

[enzo.terreno@unito.it](mailto:enzo.terreno@unito.it)

**Materials and Methods**

**Calculation Rationale of the liposome concentration:**

- [F]_tot_​: concentration of PF₆⁻ in the suspension
- [F]_lipo_: concentration of PF₆⁻ inside the liposomes (assumed to be the hydration concentration)
- V_susp_: volume of the liposome suspension after dyalisis
- V_intralipo_ ​: total intraliposomal volume
- V_lipo_ ​: aqueous core volume of a single liposome = $\frac{4}{3}\pi{r_{intralipo}^{3}}$,

where r_intralipo_ = [(d_liposome_)/2]- 5 nm (5 nm is the estimated bilayer thickness)

- [lipo]: liposome concentration in the suspension
- N_Av_​: Avogadro's number

Assuming all PF₆⁻ is encapsulated inside the liposomes:

[F]_tot_ x V_susp_=[F]_lipo_ x V_intralipo_

Solving for the total internal volume:

$$V_{intralipo}= \frac{{[F]}_{tot}}{{[F]}_{lipo}} x V_{susp}$$

The number of liposomes is then:


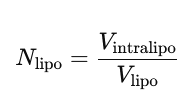


And the molar concentration of liposomes is:


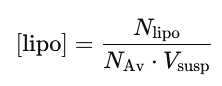


Combining everything into a single formula:

$$\left[ lipo \right]= \frac{{[F]}_{tot}{x V}_{susp}}{{[F]}_{lipo} x V_{lipo}x N_{Av} x V_{susp}}$$

V_susp_​ appears both in the numerator and the denominator at intermediate steps. This means it can be canceled out (simplified), and does not appear in the final formula.

**Results**

**Figure S1:** Comparison between the ^19^F relaxation times, T_1_ (a) and T_2_ (b) of PF_6_^-^ at different concentrations: non encapsulated (grey),and encapsulated (black) in liposomes (7 T, room temperature).

**^31^P T_1_ and T_2_ relaxation times of PF_6_^-^-loaded liposomes**

^31^P T_1_ and T_2_ relaxation times were determined using conventional inversion recovery (IR) and Carr-Purcell-Meiboom-Gill (CPMG) sequences, respectively. Data were recorded using a Bruker Avance 600 MHz NMR spectrometer. The following acquisition parameters were used: for the IR sequence, D1=20-35 s, 18 points, NS=16-256. For the CPMG sequence, the parameters were D1=35 s, NS=16, and 18 points.

The values of T1 and T2 relaxation times are reported in Table S1.

|  | T_1_ (s) | T_2_ (ms) |
| --- | --- | --- |
| FreePF_6_^-^ 150mM | 5.49 ± 0.09 | 967 ± 22 |
| LipoPF_6_^-^ 150mM | 2.86 ± 0.09 | 149 ± 10 |
| LipoPF_6_^-^ 100mM | 2.88 ± 0.08 | 177 ± 15 |
| LipoPF_6_^-^ 50mM | 4.83 ± 0.35 | 293 ±28 |

**Table S1:** ^31^P T_1_ and T_2_ relaxation times of PF_6_^-^-loaded liposomes at different concentrations and of free PF_6_^-^ 150mM.


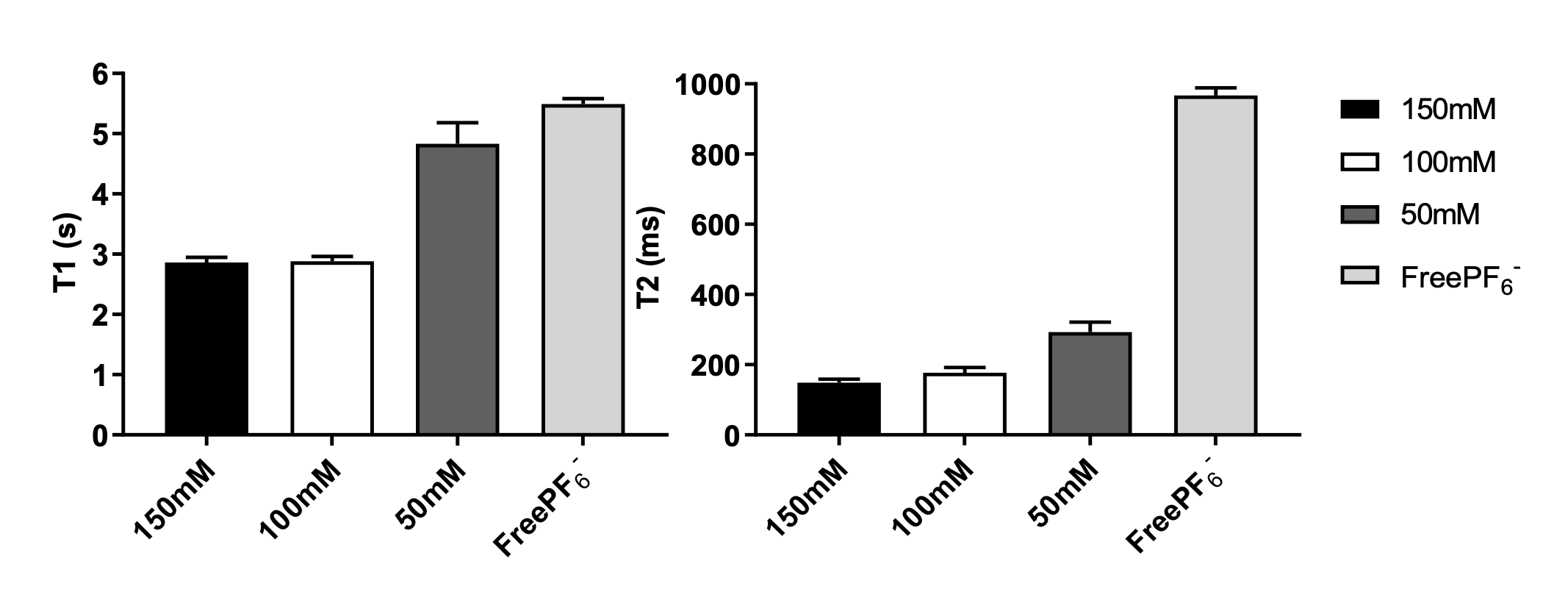


**Figure S2:** ^31^P T_1_ and T_2_ relaxation times of liposomes encapsulating PF_6_^-^ at different concentrations compared to a free PF_6_^-^ solution 150mM.


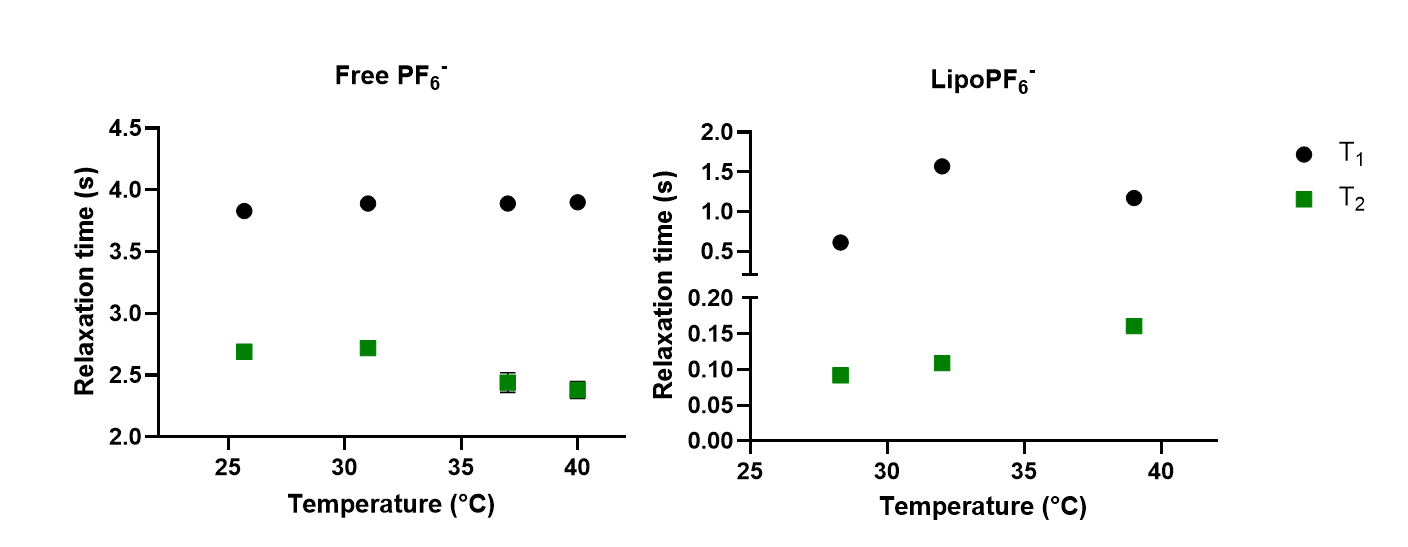


**Figure S3.** ^19^F T_1_ and T_2_ measurements of freePF_6_^-^ and LipoPF_6_^-^ 150mM at different temperatures.


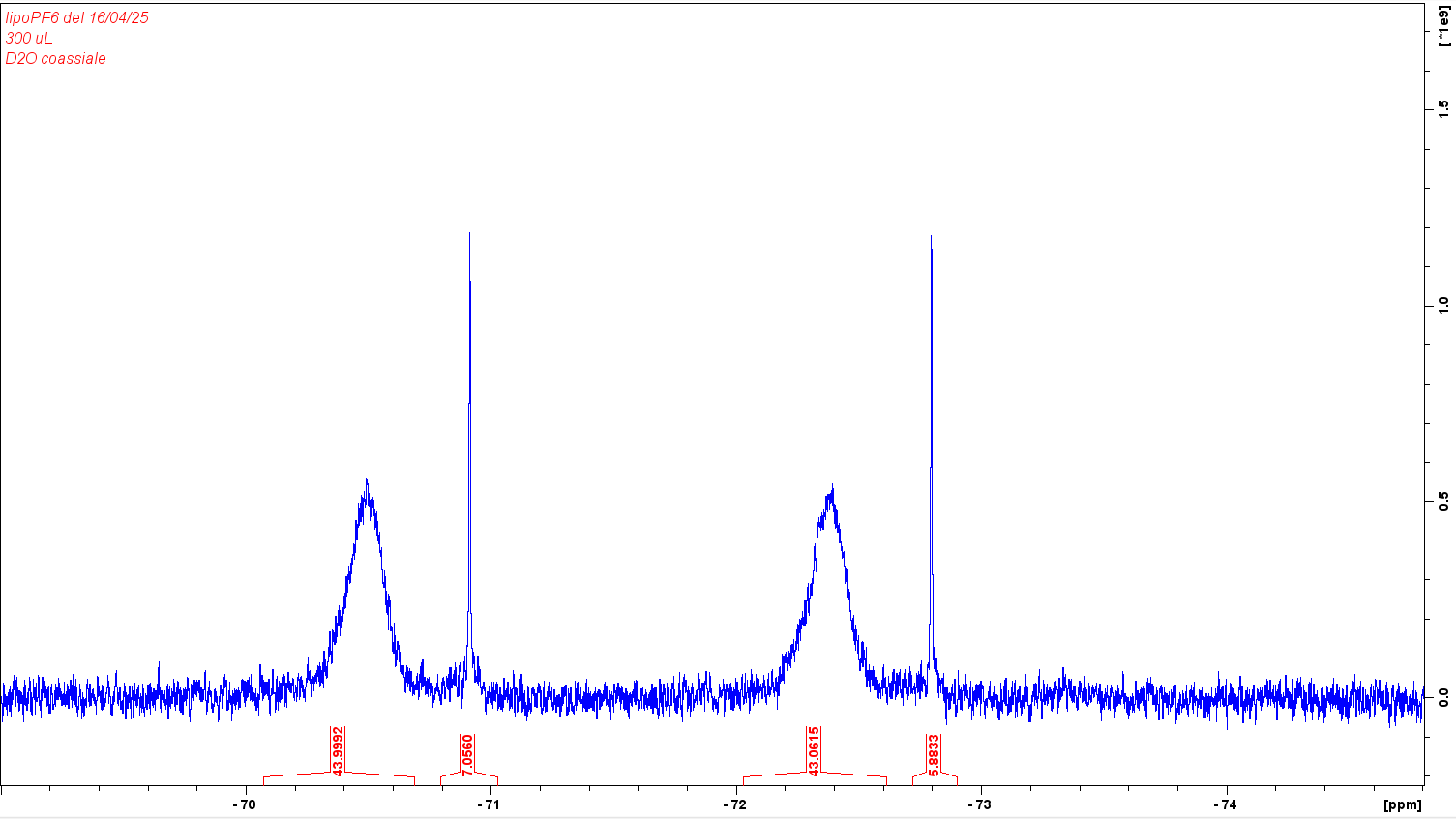


**Figure S4**. ^19^F NMR spectrum of small-sized liposomes (70 nm) acquired at 400 MHz, 298 K, 4 hours after dialysis, demonstrating the simultaneous presence of both internalized (larger peaks) and released NaPF₆.

|  | Free PF_6_^-^ | PF_6_^-^ + Lipo | LipoPF_6_^-^ |
| --- | --- | --- | --- |
| T_1_ (ms) | 3600 ± 80 | 2727 ± 106 | 382 ± 14 |
| T_2_ (ms) | 2500 ± 20 | 352 ± 9.9 | 9.4 ± 0.7 |

**Table S2:** ^19^F T_1_ and T_2_ relaxation times values of free PF_6_^-^ (150 mM), liposomes encapsulating PF_6_^-^ (PF_6_^-^ inside) and liposomes added with PF_6_^-^ (PF_6_^-^ outside) (7 T, room temperature).

|  | Free NaF | LipoNaF |
| --- | --- | --- |
| **T_1_ (ms)** | 1330 ± 10 | 680 ± 30 |
| **T_2_ (ms)** | 562 ± 7 | 94 ± 7 |

**Table S3:** ^19^F Longitudinal (T_1_) and transverse (T_2_) relaxation times of a 150 mM solution of fluoride ion and of a suspension of liposomes encapsulating 150 mM of fluoride ion (7 T, room temperature).

|  | 140 nm | 400 nm |
| --- | --- | --- |
| T_1_ (ms) | 382 ± 14 | 2400 ± 29 |
| T_2_ (ms) | 9.4 ± 0.7 | 296 ± 11 |

**Table S4:** ^19^F T_1_ and T_2_ relaxation times values of liposomes encapsulating PF_6_^-^ with a hydrodynamic diameter of 140 and 400 nm.
